# Investigating Everyday Musical Interaction During COVID-19: An Experimental Procedure for Exploring Collaborative Playlist Engagement

**DOI:** 10.3389/fpsyg.2021.647967

**Published:** 2021-04-01

**Authors:** Ilana Harris, Ian Cross

**Affiliations:** ^1^Department of Education and Psychology, Center for Cognitive Neuroscience Berlin, Free University Berlin, Berlin, Germany; ^2^Faculty of Music, Centre for Music and Science, University of Cambridge, Cambridge, United Kingdom

**Keywords:** musical interaction, virtual, social, technologically-mediated, empathy

## Abstract

Musical Group Interaction (MGI) has been found to promote prosocial tendencies, including empathy, across various populations. However, experimental study is lacking in respect of effects of everyday forms of musical engagement on prosocial tendencies, as well as whether key aspects—such as physical co-presence of MGI participants—are necessary to enhance prosocial tendencies. We developed an experimental procedure in order to study online engagement with collaborative playlists and to investigate socio-cognitive components of prosocial tendencies expected to increase as a consequence of engagement. We aimed to determine whether mere *perceived* presence of a partner during playlist-making could elicit observable correlates of social processing implicated in both MGI and prosocial behaviors more generally and identify the potential roles of demographic, musical, and inter-individual differences. Preliminary results suggest that for younger individuals, some of the social processes involved in joint music-making and implicated in empathic processes are likely to be elicited even by an assumption of virtual co-presence. In addition, individual differences in styles of listening behavior may mediate the effects of mere perceived partner presence on recognition memory.

## Introduction

The COVID-19 pandemic has brought about locally and governmentally imposed quarantine measures across the globe, resulting in prolonged social isolation. Long-lasting detriment to public mental health is a reported consequence of critical importance (Brooks et al., 2020; Gonçalves et al., 2020). In the past decade, research has shown that various forms of musical engagement with others can positively impact on an individual's socioemotional well-being; this has been reported as an outcome of both specific interventions and other forms of participatory music-making (Hallam, 2010; Hallam et al., 2014; Wilson and MacDonald, 2019; Perkins et al., 2020) as well as of particular familial and cultural contexts on listening behavior (Packer and Ballantyne, 2011; Boer and Abubakar, 2014). More recently, qualitative research on group musical engagement during the COVID-19 pandemic has suggested that involvement in online musical interaction in educational and improvisational settings may positively impact on individuals' psychological well-being and communities' connectedness (de Bruin, 2021; MacDonald et al., 2021).

A form of engagement that can be termed Musical Group Interaction (MGI), when two or more individuals make music together, can have the capacity to bring about positive consequences for group well-being (e.g., encouraging cooperation), and, remarkably, has also been found to promote domain-general empathic tendencies (Kirschner and Tomasello, 2010; Rabinowitch et al., 2013). It has been postulated that prosocial transfer effects resulting from MGI arise from its underlying mechanisms activating those which are also implicated in prosocial behaviors (Cross et al., 2012; Rabinowitch et al., 2013).

When people engage in participatory music-making (an overarching form of musical engagement encompassing MGI—for a fuller account see Turino, 2008), they are interacting with each other toward a common goal that is primarily social (Cross and Woodruff, 2009; Koelsch, 2010). To facilitate the “togetherness” predicated by this shared yet tacitly determined goal, MGI invokes a set of core components among its participants which are grounded in processes of social cognition (i.e., Empathy Promoting Musical Components (EPMCs), outlined by Cross et al., 2012). Some of these components, such as disinterested pleasure, floating intentionality, and semantic ambiguity (Kant, 1951; Cross, 1999; Pearce and Rohrmeier, 2012), arise due to communicative properties inherent to the medium of music, while others pertain to those behaviors specific to *group* engagement in general, such as shared intentionality and intersubjectivity (Trevarthen and Aitken, 2001; Cross et al., 2012). In line with the dual process framework theorized to underscore social cognition (Happé et al., 2017), EPMCs are thought to rest on both cognitive and affective mechanisms elicited during MGI, including affective alignment and cognitive co-representation amongst co-performers (Knoblich et al., 2011; Rabinowitch et al., 2013). It is hypothesized that fundamental neurocognitive mechanisms such as entrainment—which may also be implicated in coordination in non-musical joint action (see, e.g., Ogden and Hawkins, 2015)—underpin the cognitive and affective dimensions of MGI (Keller, 2014), though it has not been empirically established which of these neurocognitive mechanisms are *necessary* in order to activate specific higher-order core components of social cognition (Happé et al., 2017). The likelihood that both social motives and functions of musical involvement underpin many, if not all, musical behaviors highlights the question of which component processes of Musical Group Interaction are most significant for social cognition (Hallam, 2002; Turino, 2008; Cross, 2014).

Cross et al. (2012) outline a musical interaction programme comprising a set of tasks, each designed to pinpoint a particular EPMC. A shared characteristic of the programme's tasks is an emphasis on musical behaviors that are “other-directed” (rather than self-directed) and “mutually interdependent” (rather than individually sustainable). Rabinowitch et al. (2013) conducted a longitudinal study implementing the musical interaction programme within an educational intervention; using validated self-report and behavioral measures, they provided empirical evidence indicating an increased propensity of participating schoolchildren to “sustain and renew ongoing musical interaction[s]” during, and to engage in prosocial behaviors subsequent to, the intervention (2013). Similar findings have since been replicated among various populations (e.g., Gerry et al., 2012; Trainor et al., 2012; Schellenberg et al., 2015). However, it is unknown whether other forms of group musical engagement, such as those which need not involve real-time simultaneous interaction, are able to elicit similar social processes or prosocial benefits. Many contemporary everyday musical behaviors, or those pertaining to musical involvement in routine, everyday life (Sloboda, 2010), incorporate variants of sequential participatory performance (e.g., karaoke, collaborative playlist-making, etc., Turino, 2009) where particular characteristics of MGI (e.g., face-to-face-ness or “real” co-presence) may be absent. Scientific study of such everyday musical behaviors may offer insight into a) how EPMCs may be beneficially identified in such quotidian and potentially “non-expert” musical activities and b) which EPMCs may be conserved in “distanced” or remote forms of MGI where co-presence is technologically mediated or even simply assumed. This area of research becomes ever more pertinent as, amidst the current pandemic, we search for new forms of remote interactive behavior that still manage to evoke the meaningful experiences and critical benefits afforded by their face-to-face originals.

In this paper, we investigate collaborative playlisting, a selection method for music listening involving two or more people, in terms of its potential as an accessible and widespread form of everyday interactive musical engagement. Our primary aim was to develop a method for studying collaborative playlist-making and -listening behaviors and to assess participants' domain-specific and domain-general socio-cognitive capacities, all within the framework of an online experiment. Additionally, we aimed to identify key demographic, musical, and inter-individual differences that might impact on participants' ability to participate in collaborative playlist-making and listening as well as their subsequent socio-cognitive dispositions. The underlying theoretical presupposition is that shared intentionality, a necessary component of face-to-face MGI whereby interlocutors prioritize shared (social) goals of the musical interaction above individual goals, may be retained in online musical interaction, and that mere *perceived* participation of an interactive partner is likely to result in increased activation of processes of shared intentionality. Our first testable hypothesis was that the socio-cognitive components of self-other overlap and cognitive perspective taking would be increased following participation in the experiment's playlist-making/-listening sessions for those participants who were prompted to interact with an online partner during the session (i.e., those in the “perceived presence of an interactive partner” experimental condition). Our second hypothesis was that there would be no differences among participant groupings based on factors including demographic, musical and inter-individual differences on self-report and behavioral assessment of socio-cognitive capacities after participation in the playlist-making and listening sessions. Although results were not clear-cut, interesting effects of participants' musical and demographic background (including factors such as age, self-identification as musician or non-musician, preference for social functions of music listening, daily listening behavior) on self-reported and behavioral indices of social processing emerge from the data. We discuss these findings in terms of their theoretical significance and outline how a future iteration of the experiment might adjust for possible confounds in the hope of stimulating further research on the considerable socio-affective potential of technologically mediated musical interaction.

## Methods

### Participants

Participants were recruited on a voluntary basis. Eligibility criteria included access to the internet and to a computer with the capacity to play sound, otherwise no further criteria (e.g., prerequisites of musical expertise or genre familiarity) were stipulated. Each participant's incentive was a 5 GBP charity donation to Arts Every Day, a non-profit organization that develops music and arts education in Baltimore City Public Schools. Consent was obtained prior to and following participation in the experiment. Participants were debriefed at the close of the experiment. Ethical approval was granted by the Ethics Committee of the University of Cambridge Faculty of Music. We recruited a total sample of (*n* = 100) in order to overshoot those sample sizes used in similar experiments investigating mere social presence in virtual settings (see Platania and Moran, 2001; Bente et al., 2007; Liu and Shrum, 2009). Eight participants who began the experiment were not able to complete it and thus their data were discarded; an additional two participants' data were excluded on the basis of outlier detection. Remaining participants' response data (*n* = 90) was cleaned and pre-processed with Python libraries *NumPy* and *pandas* (Mckinney, 2010; Harris et al., 2020).

### Stimuli

30 10-s clips of instrumental hip-hop songs (details available in Supplementary Material) were used as musical stimuli in an effort to shed empirical light on the therapeutic potential of interventions using hip-hop music (see Gold et al., 2017; Crooke, 2018; Crooke and Mcferran, 2019).

### Experimental Design

Participants were asked to complete a questionnaire (see Supplementary Material) assessing demographic and musical backgrounds and assigned to either an algorithm (ALG) or a fake partner (FP) condition to which they were blind. Questions addressed familiarity with hip-hop music (5-point Likert), preference for social functions of music listening (two items; both 5-point Likert), and quantity of music listened to per day (5-point scale ranging from <1 to ≥7 h/day). Participants were told that they would be creating three playlists that were to be used at a future virtual social event (i.e., “imagine you have plans to e-meet with some friends later this week, and they have asked you to make playlists you all can listen to while you hang out”). Participants in the FP condition were told that they would be making playlists along with another participant (in fact, a fake partner), whose name, age, and place of residence was matched to those of the participant and provided to the participant via a text-only prompt along with a generic non-moving digital avatar (i.e., gender/ethnicity non-specific). Participants in the ALG condition were told that they would be making such playlists on their own, but that they would have the assistance of MusicBot, a recommendation algorithm, to which a generic non-moving digital logo was assigned and provided to the participants.

The experimental structure involved trials each of one Playlist-Making session, one intermediate “series of prompts” step (i.e., intended to elicit either perceived interaction or no perceived interaction for participants in the FP or ALG experimental condition, respectively), and one Playlist-Listening session. Each participant took part in one trial for each of the three playlists. For each Playlist-Making session, participants were told to listen through a list of 10 song clips provided by the experimenter and to select, in any order, three of those clips to add to the playlist (i.e., based on those clips that the participant liked and thought sounded good together). Subsequent to each Playlist-Making session, participants were told via a series of prompts that either another participant (FP) or a song recommendation algorithm (ALG) had added three additional clips to each playlist (in reality, clip additions were random). Finally, participants took part in a Playlist-Listening session, during which they listened to the shuffled playback of their resultant playlist (i.e., the six songs that had resulted from their song clip selections from the most recent playlist-making session along with the FP/ALG's subsequent clip additions); participants in the FP condition were prompted to engage in *joint* listening behavior (i.e., “listen with [respective fake partner name] to the playlist you both have created”), whereas participants in the ALG condition were not (i.e., “listen to the playlist that has been created”).

Following the experimental trials, a recognition task was used in order to estimate cognitive self-other overlap, in which participants' memory of clips from Playlist-Listening sessions was measured. Participants were played a randomly selected song clip from the Playlist-Making sessions and prompted to identify whether the song clip had also appeared in a playlist during any of the Playlist-Listening Sessions. All clips had previously been heard by participants during the Playlist-Making sessions (see Supplementary Material for a recognition task trial example), but the first and last 500 ms of each clip were removed to control for immediate recognition (Schellenberg et al., 1999; Filipic et al., 2010; Belfi et al., 2018). Participants were prompted to respond using a keypress as to whether the song clip corresponded to one of their own previous playlist additions, one of the FP/ALG's previous playlist additions, or had not occurred in any of the previous playlists. A total of five practice trials and 25 test trials were administered. Finally, participants were prompted to answer self-report items assessing self-other overlap (i.e., “Which picture best describes your relationship with [fake partner name]/Musicbot?”; inclusion of other in self, via IOS) and domain-general indices of trait empathy relevant to the first hypothesis (i.e., perspective-taking subscale of the Interpersonal Reactivity Index; see Supplementary Material for exact items used). The experiment was conducted online and written using PsychoJS (Peirce, 2007) by the first author.

### Participant Groups

Following data collection, participants were grouped categorically for each of the following independent variables: experimental condition (*cond*, ALG:0, FP:1); age (*age*, <=25:0, >25:1); gender (*gen*, 0:female, 1:non-binary, 2:male); musical background (*mus_back*, nonmusician:0, musician:1); hours per day spent listening to music (*hrs_list*, <1–3 h:0, ≥3–4 h:1); hip-hop genre familiarity (*hh_fam*, Likert with ≤3:0, >3:1); and two measures of the extent to which participants privileged social functions in music listening (“social relatedness” as described in Schäfer et al., 2013), *mus_soc1* and *mus_soc2*, (both Likert with ≤3:0, >3:1). Variables that we felt should shed light on participants' individual differences related to demographic and musical background were selected for analysis in order to quantitatively assess the potential for collaborative playlisting to constitute *everyday* musical interaction (i.e., that which should be minimally influenced by individual musical differences) and also to gain insight on the extent to which *demographic* factors would influence the degree to which prosocial transfer effects might result.

## Results

### Self-Report Analyses

**Figure 1:**
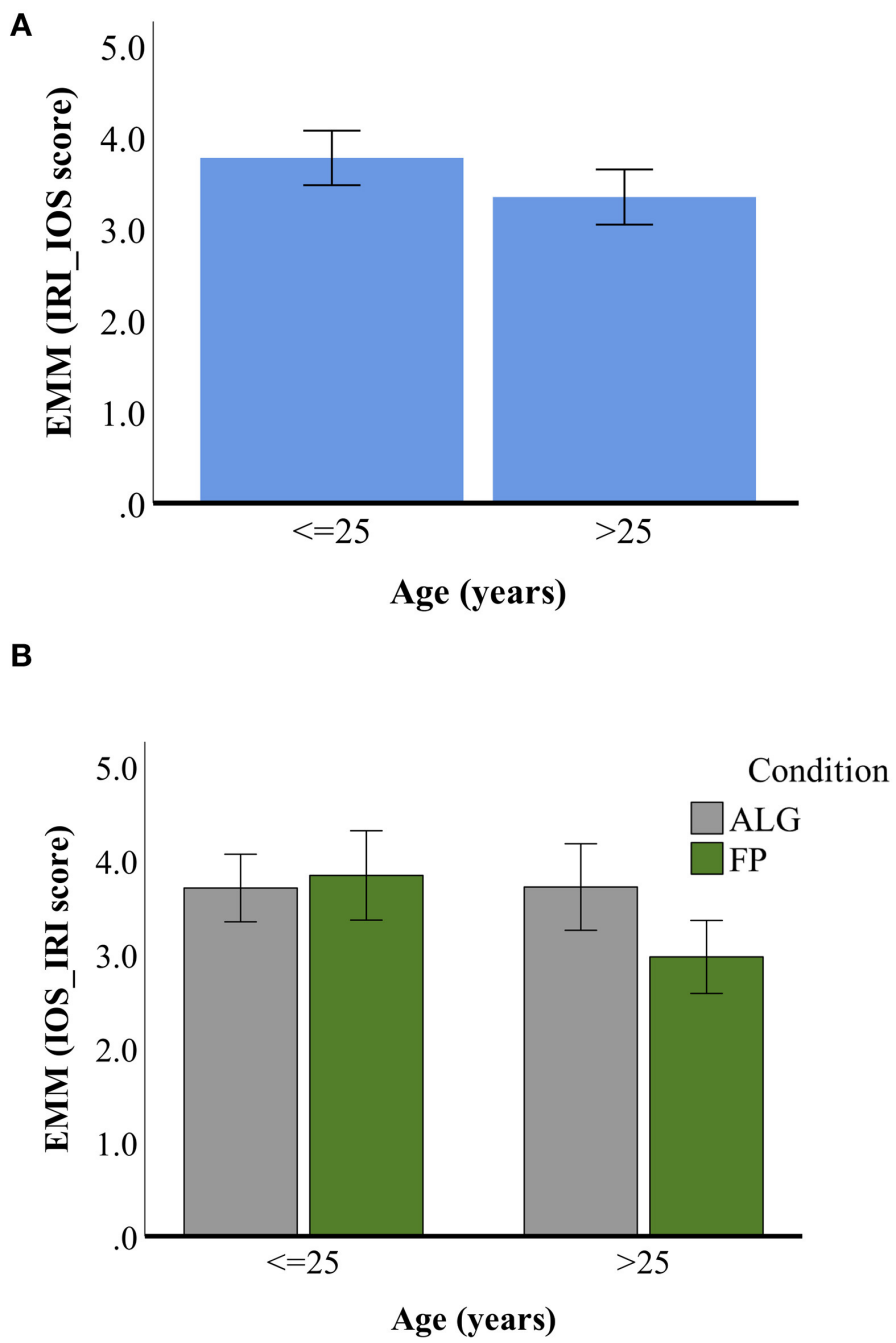
Estimated Marginal Means (EMM) of composite IOS_IRI score (IOS_IRI score) for between-subject effects of **(A)** *age*, with EMMs for *n* = 43 participants <=25 years of age and *n* = 47 participants >25 years of age; **(B)** age*cond, gray bars denoting EMMs for participants in the ALG experimental condition and green bars for participants in the FP condition. For each panel, Error Bars correspond to 95% confidence interval.

A composite dependent variable containing the average of participant's responses to IOS and IRI self-report items was used to determine participants' self-reported prosocial tendencies following the experiment [*CV_iosiri*; continuous with range (1.5, 6.0) inclusive]. Participant groupings based on *gen, hrs_list, hh_fam* and *mus_soc2* did not have normal distributions between-subjects for the composite self-report dependent variable and were excluded from further analyses. A factorial ANOVA (full results in Supplementary Material) with the remaining variables (*cond, age, mus-back*, and *mus_soc1*) was conducted to explore differences between participants' composite self-report prosocial tendencies scores. A significant main effect for *age* was found *F*_(15, 74)_ = 4.06, *p* = 0.048; Figure 1A. Significant interaction effects for age^*^cond (Figure 1B) and mus_back^*^ *mus_soc1* were also found *F*_(15, 74)_ = 4.26, *p* = 0.043 and *F*_(15, 74)_ = 4.78, *p* = 0.014, respectively. *Post-hoc* comparisons using the Tukey HSD test showed that the mean score for participant groupings *age* ≤ *25* was significantly different from *age* > *25* in the FP experimental condition (Mean Difference = 0.826, SE = 0.623, *p* = 0.012). The interaction effect of mus_back^*^ *mus_soc1* was disordinal and therefore not able to be further interpreted.

Results of our analyses for the self-report data suggest that age (i.e., whether an individual is in early adulthood/adolescence or is in middle/late adulthood) impacts on self-reported prosocial tendencies following online engagement in playlist-making/-listening behavior. Furthermore, our analyses suggest that age also impacts on self-reported prosocial tendencies following online engagement in *perceived collaborative* playlist-making/-listening, with individuals in middle/late adulthood tending to report lower prosocial tendencies following perceived collaborative playlist-making/-listening.

### Behavioral

#### Task Analyses

Participants were grouped categorically for (*cond, age, mus-back*, and *mus_soc1*). Two within-subject conditions were identified, the first corresponding to those song clips that had either been selected by the participant or by the FP/ALG for any playlists (“no self-other overlap”), the second corresponding to those song clips that had been selected by *neither* for any playlist (“full self-other overlap”). These conditions were used in order to be comparable to those proposed by Aron and Fraley (1999). Initially, sensitivity scores (d') for each participant across all “no self-other overlap” and “full self-other overlap” task items were calculated. However, 18 participants showed either ceiling or floor effects for “no self-other overlap” items. Hence hit rate was used as the dependent variable in order to preserve as much data as possible in our already small sample size, where the ratio of hits/hits+misses was determined for “no self-other overlap” and “full self-other overlap” task items.

**Figure 2:**
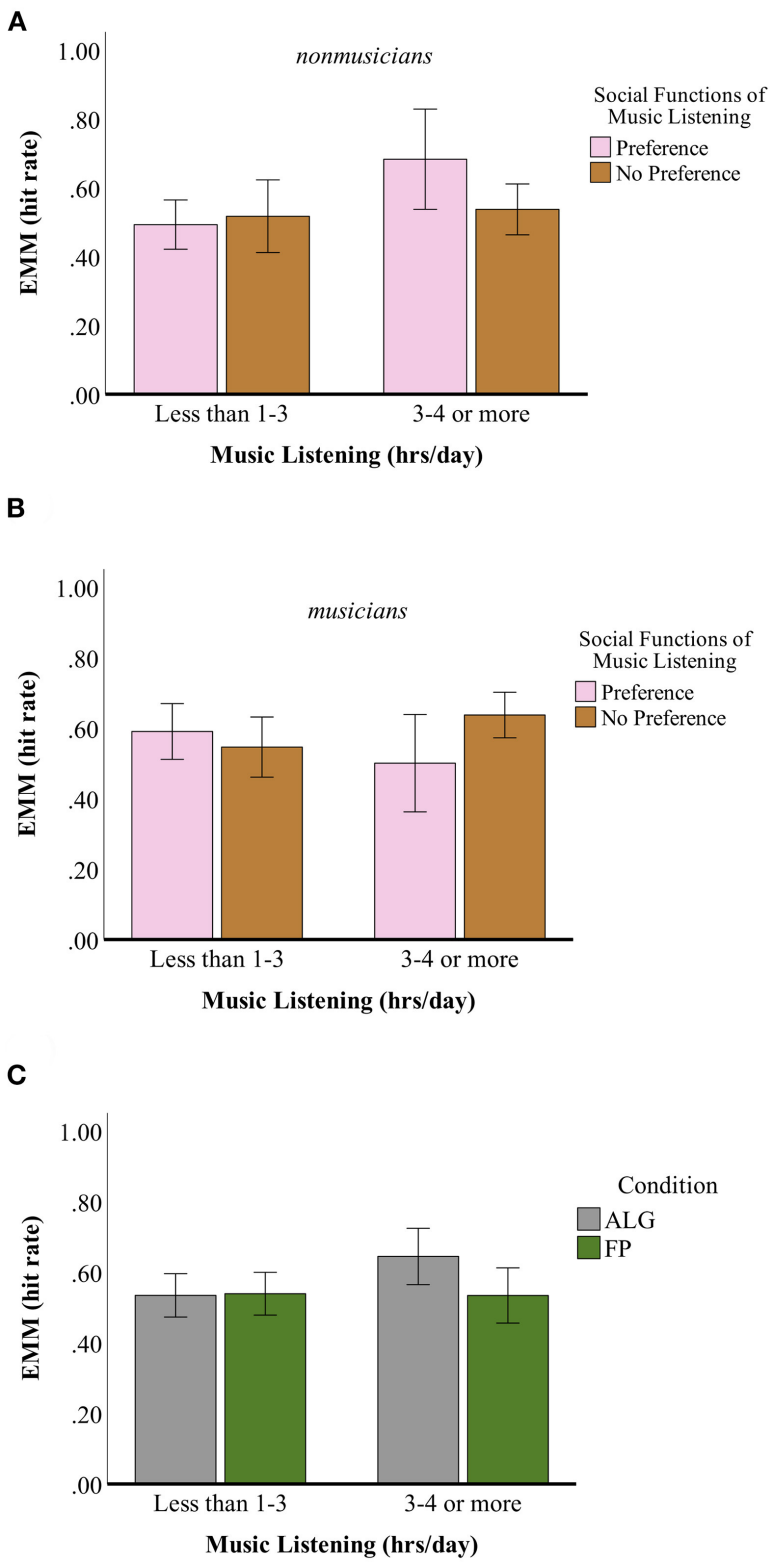
Estimated Marginal Means (EMM) of overall hit rate during recognition task (hit rate) for between-subject effects of mus_back*hrs_list*mus_soc1, with **(A)** vs. **(B)** panels showing EMMs for non-musicians vs. musicians (*mus_back* = *0 vs. mus_back* = *1*); pink bars denoting EMMs for participants with scores <=3 for *preference for social functions of music listening* item, brown bars for scores of >3 (*mus_soc1*); and x-axis separating EMMs for participants who reported either listening to ≤1–3 or ≥3–4 h of music per day **(C)** cond*hrs_list, with gray bars denoting EMMs for participants in the ALG experimental condition and green bars in the FP condition. For each panel, Error Bars correspond to 95% confidence interval.

A mixed, repeated measures ANOVA (full results in Supplementary Material) was conducted to determine whether any significant differences between groups of participants existed for performance during the recognition task (assessed via hit rate) on the basis of task item type (i.e., “no self-other overlap” or “full self-other overlap” conditions). Participant groupings based on between-subject variables *age, gen, hh_fam*, and *mus_soc2* did not have normally distributed hit rate data and thus were excluded from further analyses. A significant between-subjects 3-way interaction effect for mus_back^*^hrs_list^*^mus_soc1 on overall hit rate (*hitrate*) was found *F*_(1, 74)_ = 6.14, *p* = 0.016; Figures 2A,B. A marginally significant interaction effect for cond^*^hrs_list was also found *F*_(1, 74)_ = 2.64, *p* = 0.11; Figure 2C. No significant within-subject differences were found. The between-subjects three-way interaction effect of mus_back^*^hrs_list^*^mus_soc1 was further assessed using a one-way ANOVA (i.e., using a grouping variable) to assess simple main-effects across the level of each independent variable which showed that: a) the interaction for mus_back^*^mus_soc1 was significant when *hrs_list* = *1* was held constant, for *p* < 0.025 (α adjusted per family error rate) and b) the interaction of mus_back^*^hrs_list was significant when *mus_soc* = *0* was held constant, for *p* < 0.025 (α adjusted per family error rate). No further significant differences were found among participant subgroups comprising the two-way interactions of mus_back^*^mus_soc1 at *hrs_list* = *1* or mus_back^*^hrs_list at *mus_soc* = *0*. No further significant differences were found among participant groupings for the interaction effect of cond^*^hrs_list.

In sum, results of our analyses for the behavioral recognition task suggest that several demographic and musical differences may interact with the potential for recognition memory of songs to shed meaningful light on cognitive self-other overlap consequent to collaborative playlist. Among individuals who listen to ≥1–3 h of music daily, an interaction between musicianship and the privileging of social functions of music appears to impinge on song recognition memory, while an interaction between musicianship and daily hours of music listening affects song recognition memory for individuals who do *not* privilege social functions of music listening. As no significant within-subject differences were found, the task in its present form is not a reliable index for cognitive self-other overlap following playlist-making/-listening, while the ceiling effect observed among several participants' within-subject data (*n* = 18) indicates that refinement of the task's difficulty level and perhaps pre-testing of potential participants would be desirable.

## Discussion

### General Limitations

The principal limitation of the experiment was a relatively small sample of participants (*n* = 90) for a relatively large number of between-group splits (eight splits); much larger sample sizes would need to be obtained for future iterations of this experiment to produce definitive findings. Given the small sample size, it would have been advantageous had baseline data for both self-report and behavioral measures been gathered prior to the experiment, which could have shed light on how the experiment itself affected participants' prosocial tendencies. In addition, baseline measures of inter-individual differences pertaining to participants' routine online engagements in non-musical contexts (i.e., daily use of social media, preferred functions of social media use) would have been advantageous in controlling for further potential confounds.

### Self-Report

Interpretation of Figure 1A in the light of the significant main effect found for age on self-reported prosocial tendencies suggests that, following online engagement with playlist-making and playlist-listening *in general*, younger participants (age 25 or younger) tend to report higher self-other overlap and trait empathy scores in comparison to older participants. This distinction between >25 and ≤25 years of age corresponds roughly to the distinction between Generation Z, the first whole-life “digital native” generation (Dimock, 2019), and older cohorts. This indication that younger participants may be more inclined to perceive interactions occurring online as being social is not surprising; in 2018 it was reported that 70% of American teens aged 13–17 check social media several times a day (Richter, 2018), whereas use of at least one social media site for adults aged 18–29, 30–49, 50–64, and 65+ was reported to be 88, 78, 64, and 37%, respectively (Pew Research Center, 2018); research has shown that opportunities afforded by online social interactions may allow young people to experiment with and form their identity (Leung, 2011). In fact, technologically mediated contexts may constitute an *integral part* of younger individuals' expectations and representations of inter-personal behavior, and positively affect the individual (e.g., decrease loneliness; Leung, 2011) as well as the individual's existing relationships (e.g., improve quality of existing friendships; Valkenburg and Peter, 2009). However, the benefits and types of social interactions (e.g., occurring with known persons as opposed to strangers) in which individuals are motivated to engage have been shown to hinge on age; moreover, individuals' baseline loneliness and existence of offline social support may mediate the benefits of social technology (Skues et al., 2012; Chopik, 2016; Nowland et al., 2018). Nonetheless, we expect that social processes implicated in online musical interaction are likely to have a higher *potential* for activation for members of Generation Z, with a differential effect on early vs. late adolescent subgroups when online musical interaction is carried out with a stranger as opposed to a known individual. Particularly for early adolescents, who have an increased risk of developing clinical mental health conditions as a consequence of social isolation (Loades et al., 2020), use of music in online interactions may help individuals to stay connected with their communities (e.g., with peers from school and extra-curricular activities) and mediate negative effects of social isolation on psychological health (Moore and March, 2020; MacDonald et al., 2021).

Figure 1B allows for more nuanced consideration of the between-subject effect of age on prosocial tendencies. It is apparent that younger participants in the perceived collaboration condition tended to report higher self-other overlap and trait empathy compared to older participants in that condition. However, the significant difference between these groups does not imply that the perceived collaboration condition was more successful in increasing self-reported prosociality in younger participants, but rather, that the perceived collaboration condition was more *unsuccessful* in increasing prosociality in the older participants. From these results we conclude the following. First, younger participants in the perceived collaboration condition may have not been amenable to their supposed partner's random playlist additions, and consequently did not report high prosocial tendencies; in fact, the random “behavior” of the supposed fake partner may have undermined the potential for tacit acknowledgment of a shared goal (necessary for fulfillment of shared intentionality), and thereby disqualified it as constituting behavior indicative of musical interaction. Future iterations of this experiment should better address this issue, for instance, by incorporating a simple machine learning algorithm (e.g., *Naive Bayes*) into determination of the fake partner's playlist additions to be more consonant with the participant's playlist additions. Moreover, an additional self-report item assessing participants' trustworthiness of the perceived collaborator could help in clarifying the potential bases for older participants' reporting of lower self-other overlap and trait empathy in the perceived collaboration condition.

### Behavioral

The possibility of understanding behavioral task results in the bigger theoretical picture of technologically mediated musical interaction and prosociality is limited by the non-existence of significant within-subject effects. Several amendments to the presented recognition task are required for future iterations of this experiment: first, a larger number of total trials should be included; second, more similar instrumental song clip stimuli should be included in the experiment in order to increase the task's difficulty level; third, control stimuli (i.e., items that did not appear *at all* during playlist-making but that would be played for participants in a separate “priming” portion of the experiment) should be included. Such revisions could increase the likelihood of our sample's data approaching normality and allow for use of more robust statistical analyses (e.g., application of the ROC curve) in order to elucidate any within-subject differences. In addition, if a similar behavioral task is to be included in future experiments, individual differences that are likely to confound overall performance and thereby hinder meaningful interpretability of the task (such as amount of music listened to per day, prioritizing social functions of music listening, and being a musician vs. non-musician) should be controlled for via the use of participant inclusion criteria.

Nevertheless, the aim of the experiment—to evaluate cognitive self-other overlap occurring as a consequence of perceived collaborative playlisting—provides a starting point for behaviorally assessing activation of socio-cognitive processes in online musical contexts. The authors are not aware of any domain-specific behavioral tasks which exist to measure cognitive indices of social processing for online musical interaction. Validated behavioral tasks assessing self-other overlap in non-musical contexts, though they are rare, might also be included in further follow-up experiments. Such triangulation of self-report and behavioral assessment of socio-cognitive processes would facilitate empirical comparison of the prosocial benefits afforded by online musical interaction vs. those afforded by face-to-face MGI, and, crucially, would allow for fine-tuning of the framework theorized to underpin the overlap between interactive musical engagement and social cognition. Furthermore, measuring behavioral indices of socio-cognitive processes involved during collaborative playlist-making may facilitate our understanding of how harm to individuals' psychological well-being resulting from prolonged social isolation may be mitigated via participation in interactive online musical engagement. Developing suitable behavioral tasks in order to be able to assess the impact of online musical interaction on psychological processes impaired by quarantine measures would contextualize findings from qualitative research (such as that which has suggested that online music improvisation may address negative effects of social isolation on psychological health; MacDonald et al., 2021) in a causal mechanistic understanding.

### Key Takeaways and Further Steps

At this stage we cannot determine which of the socio-cognitive processes activated during MGI may also be sufficiently activated by perceived presence of a partner during online collaborative playlisting. However, we are able to narrow our underlying hypothesis that shared intentionality is likely to be preserved in online musical interactions, in terms of specifying certain subpopulations to which it is likely to apply. Given the implementation of an experimental setup that is able to meet the conditions for shared intentionality (i.e., one where a perceived collaborator behaves in such a way that their working toward a goal shared by the participant is implied) and participant criterion controlling for listening behavior, we would expect that younger participants are likely to show enhanced social processing and tendency for prosocial behavior following online musical interaction—even one that is sequential. The main scientific contributions of our findings are thus: a) that researchers wishing to study social transfer effects of online music-making can do so via a collaborative playlist-making paradigm provided that requisite aspects of musical interaction are met (i.e., determination of a shared social goal and apparent contribution of interlocutors to this social goal); and b) that participant criteria should select for adolescents and young adults whose everyday listening behaviors are maximally invariable (i.e., with respect to quantity of music listening per day and preference for social functions of music listening). Future research operationalizing collaborative playlist-making paradigms should further investigate the differential effect that joint music listening may have on socio-cognitive components of prosociality. Individual music-listening has been shown to act as a social surrogate (i.e., to serve as temporary substitutes for direct social interaction; Schäfer and Eerola, 2020), to evoke implicit affiliation among listeners with high baseline trait empathy (Vuoskoski et al., 2017), and to positively impact on individuals' psychological health and life satisfaction during the COVID-19 pandemic (Bu et al., 2020; Krause et al., 2021); a controlled experiment investigating the effect of joint vs. individual music listening on functions served by music as a social surrogate as well as on implicit affiliation mediated by baseline inter-individual difference would be useful in further distinguishing the likely effects that collaborative playlist-making/-listening has on an individual's social capacities amidst social isolation. In the midst of the current global pandemic it would be of considerable benefit to attain a better understanding of which socio-cognitive consequences of musical interaction might be preserved in technologically mediated contexts, given that an increased prevalence of such modes of interaction is likely to be one lasting effect of COVID-19.

## Data Availability Statement

The original contributions presented in the study are included in the article/Supplementary Material, further inquiries can be directed to the corresponding author/s.

## Ethics Statement

The studies involving human participants were reviewed and approved by The Faculty of Music Ethics Committee at the University of Cambridge. The patients/participants provided their written informed consent to participate in this study.

## Author Contributions

IH designed and implemented the online experiment, cleaned and analyzed the data, and produced an initial version of the paper as part of her MPhil research at Cambridge, supervised by IC. IC assisted with argument development and theoretical contextualization. All authors co-authored the final version of the manuscript.

## Conflict of Interest

The authors declare that the research was conducted in the absence of any commercial or financial relationships that could be construed as a potential conflict of interest.
