# Supplementary material for: Investigating Everyday Musical Interaction During COVID-19: An Experimental Procedure for Exploring Collaborative Playlist Engagement
Source: Front Psychol. 2021 Apr 1;12:647967. doi: 10.3389/fpsyg.2021.647967 (PMC8049501; doi:10.3389/fpsyg.2021.647967)
Supplement: Supplementary file 1 [file Table_1.DOCX]

Supplementary Material

# Supplementary Data

## Frequency Tables for Questionnaire Data

### Demographics (0-valued options not shown)

| **Item** | **Original Scale** | **Raw Frequencies (n)** | **Recoded Categories** | **Recoded Frequencies (n)** |
| --- | --- | --- | --- | --- |
| Age | 1=<18 | 3 | 0 | 67 |
|  | 2=18-24 | 44 |  |  |
|  | 3=25-30 | 20 |  |  |
|  | 4=30-40 | 13 | 1 | 23 |
|  | 5=40-55 | 8 |  |  |
|  | 6=>55 | 2 |  |  |
| Gender | 0=Female | 37 | N/A | N/A |
|  | 1=Non-binary | 7 |  |  |
|  | 1=Male | 46 |  |  |
| Continent of Residence | 2=North America | 68 | N/A | N/A |
|  | 4=Europe | 18 |  |  |
|  | 3=Asia | 2 |  |  |
|  | 1=Africa | 1 |  |  |
|  | 7=Prefer not to say | 1 |  |  |
| Ethnicity | 1=Asian | 12 | N/A | N/A |
|  | 2=Black | 4 |  |  |
|  | 3=White | 49 |  |  |
|  | 4=Hispanic/Latinx | 3 |  |  |
|  | 7=None of the above | 4 |  |  |
|  | 9=Mixed Race | 18 |  |  |

### Pre-Paradigm Self-Report (data from unanalyzed items not shown)

| **Item** | **Original Scale** | **Raw Frequencies (n)** | **Recoded Categories** | **Recoded Frequencies (n)** |
| --- | --- | --- | --- | --- |
| Musical Background | 1 = (non-musician) | 10 | 0 | 59 |
|  | 2 = music-loving nonmusician | 31 |  |  |
|  | 3 = amateur musician | 18 |  |  |
|  | 4 = serious amateur musician | 17 | 1 | 31 |
|  | 5 = semiprofessional musician | 6 |  |  |
|  | 6 = (professional musician) | 8 |  |  |
| Hours Listening to Music Per Day | 1 = <1 hour | 19 | 0 | 47 |
|  | 2 = 1-3 hours | 28 |  |  |
|  | 3 = 3-5 hours | 24 | 1 | 43 |
|  | 4 = 5-7 hours | 14 |  |  |
|  | 5 = >7 hours | 5 |  |  |
| Familiarity with Hip-Hop (Likert) | 1 | 4 | 0 | 51 |
|  | 2 | 18 |  |  |
|  | 3 | 29 |  |  |
|  | 4 | 23 | 1 | 39 |
|  | 5 | 16 |  |  |
| Preference for Social Functions of Music Listening - Item 1 (Likert) | 1 | 5 | 0 | 39 |
|  | 2 | 14 |  |  |
|  | 3 | 20 |  |  |
|  | 4 | 30 | 1 | 51 |
|  | 5 | 21 |  |  |
| Preference for Social Functions of Music Listening - Item 2 (Likert) | 1 | 6 | 0 | 51 |
|  | 2 | 14 |  |  |
|  | 3 | 31 |  |  |
|  | 4 | 21 | 1 | 39 |
|  | 5 | 18 |  |  |

### Post-Paradigm Self-Report (data from unanalyzed items not shown)

| **Item** | **Scale** | **Raw Frequencies (n)** |
| --- | --- | --- |
| Interpersonal Reactivity Index (Likert) | 1 | 3 |
|  | 2 | 6 |
|  | 3 | 11 |
|  | 4 | 34 |
|  | 5 | 36 |
| Inclusion of Other in the Self Scale (Pictorial) | 1 = No self-other overlap | 16 |
|  | 2 | 14 |
|  | 3 | 21 |
|  | 4 | 24 |
|  | 5 | 12 |
|  | 6 | 2 |
|  | 7 = Full self-other overlap | 1 |

## Full Reports of Between-Subject Effects

### Between-Subject Effects: IOS and IRI Self-Report Items

| Dependent Variable: | Composite IRI-IOS score |  |  |  |  |  |
| --- | --- | --- | --- | --- | --- | --- |
| Source | Type III Sum of Squares | df | Mean Square | F | Sig. | Partial Eta Squared |
| Corrected Model | 20.994^a^ | 15 | 1.400 | 1.856 | 0.042 | 0.273 |
| Intercept | 842.778 | 1 | 842.778 | 1117.774 | 0.000 | 0.938 |
| cond | 1.550 | 1 | 1.550 | 2.055 | 0.156 | 0.027 |
| age | 3.059 | 1 | 3.059 | 4.057 | 0.048 | 0.052 |
| mus_back | 0.408 | 1 | 0.408 | 0.541 | 0.464 | 0.007 |
| mus_soc1 | 0.528 | 1 | 0.528 | 0.700 | 0.405 | 0.009 |
| cond * age | 3.209 | 1 | 3.209 | 4.257 | 0.043 | 0.054 |
| cond * mus_back | 0.073 | 1 | 0.073 | 0.097 | 0.756 | 0.001 |
| cond * mus_soc1 | 0.412 | 1 | 0.412 | 0.546 | 0.462 | 0.007 |
| age * mus_back | 1.174 | 1 | 1.174 | 1.557 | 0.216 | 0.021 |
| age * mus_soc1 | 0.010 | 1 | 0.010 | 0.013 | 0.908 | 0.000 |
| mus_back * mus_soc1 | 4.775 | 1 | 4.775 | 6.333 | 0.014 | 0.079 |
| cond * age * mus_back | 0.997 | 1 | 0.997 | 1.322 | 0.254 | 0.018 |
| cond * age * mus_soc1 | 0.520 | 1 | 0.520 | 0.689 | 0.409 | 0.009 |
| cond * mus_back * mus_soc1 | 0.814 | 1 | 0.814 | 1.079 | 0.302 | 0.014 |
| age * mus_back * mus_soc1 | 0.051 | 1 | 0.051 | 0.067 | 0.796 | 0.001 |
| cond * age * mus_back * mus_soc1 | 0.980 | 1 | 0.980 | 1.300 | 0.258 | 0.017 |
| Error | 55.794 | 74 | 0.754 |  |  |  |
| Total | 1236.000 | 90 |  |  |  |  |
| Corrected Total | 76.789 | 89 |  |  |  |  |
| a. R Squared = .273 (Adjusted R Squared = .126) | | | | | | |

#### Post-Hoc Tests for Multiple Comparisons: IOS and IRI Self-Report Items

| Tukey HSD | composite DV of ios and iri |  |  |  | 95% Confidence Interval | |
| --- | --- | --- | --- | --- | --- | --- |
|  |  |  |  |  |  |  |
|  |  | Mean Difference (I-J) | Std. Error | Sig. | Lower Bound | Upper Bound |
| (I) Grouping | (J) Grouping |  |  |  |  |  |
| 1 | 2 | -0.226 | 0.2598 | 0.821 | -0.906 | 0.455 |
|  | 3 | 0.012 | 0.2696 | 1.000 | -0.694 | 0.719 |
|  | 4 | 0.601 | 0.2598 | 0.103 | -0.080 | 1.281 |
| 2 | 1 | 0.226 | 0.2598 | 0.821 | -0.455 | 0.906 |
|  | 3 | 0.238 | 0.2723 | 0.818 | -0.475 | 0.951 |
|  | 4 | .826^*^ | 0.2626 | 0.012 | 0.138 | 1.514 |
| 3 | 1 | -0.012 | 0.2696 | 1.000 | -0.719 | 0.694 |
|  | 2 | -0.238 | 0.2723 | 0.818 | -0.951 | 0.475 |
|  | 4 | 0.588 | 0.2723 | 0.143 | -0.125 | 1.301 |
| 4 | 1 | -0.601 | 0.2598 | 0.103 | -1.281 | 0.080 |
|  | 2 | -.826^*^ | 0.2626 | 0.012 | -1.514 | -0.138 |
|  | 3 | -0.588 | 0.2723 | 0.143 | -1.301 | 0.125 |
| Based on observed means.  The error term is Mean Square(Error) = .793. | | | | | | |
| *. The mean difference is significant at the .05 level.  Participant Groupings Key:  Grouping 1: age≤25 & cond=ALG  Grouping 2: age≤25 & cond=FP  Grouping 3: age>25 & cond=ALG  Grouping 4: age>25 & cond=FP | | | | | | |

### Between-Subject Effects: Hit Rate During Recognition Task

| Dependent Variable: | Overall Hit Rate |  |  |  |  |  |
| --- | --- | --- | --- | --- | --- | --- |
|  |  |  |  |  |  |  |
| Source | Type III Sum of Squares | df | Mean Square | F | Sig. | Partial Eta Squared |
| Intercept | 32.365 | 1 | 32.365 | 1009.343 | 0.000 | 0.932 |
| mus_back | 0.003 | 1 | 0.003 | 0.097 | 0.757 | 0.001 |
| hrs_list | 0.071 | 1 | 0.071 | 2.208 | 0.142 | 0.029 |
| mus_soc1 | 0.001 | 1 | 0.001 | 0.043 | 0.837 | 0.001 |
| cond | 0.072 | 1 | 0.072 | 2.233 | 0.139 | 0.029 |
| mus_back * hrs_list | 0.070 | 1 | 0.070 | 2.178 | 0.144 | 0.029 |
| mus_back * mus_soc1 | 0.073 | 1 | 0.073 | 2.277 | 0.136 | 0.030 |
| mus_back * cond | 0.007 | 1 | 0.007 | 0.217 | 0.643 | 0.003 |
| hrs_list * mus_soc1 | 0.000 | 1 | 0.000 | 0.006 | 0.940 | 0.000 |
| hrs_list * cond | 0.085 | 1 | 0.085 | 2.644 | 0.108 | 0.034 |
| mus_soc1 * cond | 0.002 | 1 | 0.002 | 0.067 | 0.797 | 0.001 |
| mus_back * hrs_list * mus_soc1 | 0.197 | 1 | 0.197 | 6.136 | 0.016 | 0.077 |
| mus_back * hrs_list * cond | 0.014 | 1 | 0.014 | 0.444 | 0.507 | 0.006 |
| mus_back * mus_soc1 * cond | 0.001 | 1 | 0.001 | 0.028 | 0.867 | 0.000 |
| hrs_list * mus_soc1 * cond | 0.000 | 1 | 0.000 | 0.012 | 0.913 | 0.000 |
| mus_back * hrs_list * mus_soc1 * cond | 0.005 | 1 | 0.005 | 0.150 | 0.699 | 0.002 |
| Error | 2.373 | 74 | 0.032 |  |  |  |

#### Post-Hoc Test for Multiple Comparisons: Hit Rate During Recognition Task

| **One-way ANOVA: Simple Main Effects** | | | | | | |
| --- | --- | --- | --- | --- | --- | --- |
| hrs_list held constant at: | hitrate |  |  |  |  |  |
|  | Source | Type III Sum of Squares | df | Mean Square | F | Sig. |
| 0 | Corrected Model | .040^a^ | 3 | 0.013 | 1.038 | 0.385 |
|  | Intercept | 12.628 | 1 | 12.628 | 993.119 | 0.000 |
|  | mus_back | 0.021 | 1 | 0.021 | 1.619 | 0.210 |
|  | mus_soc1 | 0.002 | 1 | 0.002 | 0.150 | 0.700 |
|  | mus_back * mus_soc1 | 0.010 | 1 | 0.010 | 0.801 | 0.376 |
|  | Error | 0.547 | 43 | 0.013 |  |  |
|  | Total | 14.513 | 47 |  |  |  |
|  | Corrected Total | 0.586 | 46 |  |  |  |
| 1 | Corrected Model | .070^b^ | 3 | 0.023 | 1.659 | 0.192 |
|  | Intercept | 9.906 | 1 | 9.906 | 705.806 | 0.000 |
|  | mus_back | 0.002 | 1 | 0.002 | 0.116 | 0.735 |
|  | mus_soc1 | 0.001 | 1 | 0.001 | 0.087 | 0.770 |
|  | mus_back * mus_soc1 | **0.059*** | 1 | 0.059 | 4.212 | 0.047 |
|  | Error | 0.547 | 39 | 0.014 |  |  |
|  | Total | 15.294 | 43 |  |  |  |
|  | Corrected Total | 0.617 | 42 |  |  |  |
| a. R Squared = .068 (Adjusted R Squared = .002) | | | | | | |
| b. R Squared = .113 (Adjusted R Squared = .045)  *p-value initially calculated by hand and assessed for significance α adjusted per family error rate. | | | | | | |

| **One-way ANOVA: Simple Main Effects** | | | | | | |
| --- | --- | --- | --- | --- | --- | --- |
| mus_soc1 held constant at: | hitrate |  |  |  |  |  |
|  | Source | Type III Sum of Squares | df | Mean Square | F | Sig. |
| 0 | Corrected Model | .073^a^ | 3 | 0.024 | 1.928 | 0.143 |
|  | Intercept | 9.284 | 1 | 9.284 | 734.008 | 0.000 |
|  | mus_back | 0.002 | 1 | 0.002 | 0.122 | 0.729 |
|  | hrs_list | 0.008 | 1 | 0.008 | 0.655 | 0.424 |
|  | mus_back * hrs_list | **0.058*** | 1 | 0.058 | 4.560 | 0.040 |
|  | Error | 0.443 | 35 | 0.013 |  |  |
|  | Total | 12.480 | 39 |  |  |  |
|  | Corrected Total | 0.516 | 38 |  |  |  |
| 1 | Corrected Model | .066^b^ | 3 | 0.022 | 1.582 | 0.206 |
|  | Intercept | 13.681 | 1 | 13.681 | 987.102 | 0.000 |
|  | mus_back | 0.021 | 1 | 0.021 | 1.538 | 0.221 |
|  | hrs_list | 0.013 | 1 | 0.013 | 0.955 | 0.334 |
|  | mus_back * hrs_list | 0.011 | 1 | 0.011 | 0.758 | 0.388 |
|  | Error | 0.651 | 47 | 0.014 |  |  |
|  | Total | 17.327 | 51 |  |  |  |
|  | Corrected Total | 0.717 | 50 |  |  |  |
| a. R Squared = .142 (Adjusted R Squared = .068) | | | | | | |
| b. R Squared = .092 (Adjusted R Squared = .034)  *p-value initially calculated by hand and assessed for significance α adjusted per family error rate. | | | | | | |

# Supplementary Information

## Full (Step-By Step) Overview of Experimental Design

1. Self-report Items (Pre):
   1. Musical background (single-item measure for identifying musicians and non-musicians from Zhang & Schubert, 2019)
   2. Music listening behavior (single-item measure for hours per day spent listening to music from North et al., 2000)
   3. Genre familiarity (adapted from Van Den Bosch et al., 2013)
   4. Preferred functions of music listening: items specific to social functions Schäfer et al., 2013)
   5. Ten-Item Personality Measure* (i.e. TIPI; Gosling et al., 2003)
   6. Positive and Negative Affect Scale* (i.e. PANAS; Mackinnon et al., 1999)
   7. Single-item measure for perceived stress* (Elo et al., 2003)
2. Demographic Questions
   1. Categorical assessment of age (e.g., “18-24 years,” 25-30 years of age,” etc.)
   2. Gender (i.e., female, male, non-gender binary)
   3. Continent of residence
3. Condition Assignment
   1. Participants assigned to either:
      1. Algorithm (ALG) condition
      2. Fake Partner (FP) condition
   2. Participants informed that ALG/FP will be assisting them with playlist-making
4. Playlist Paradigm (Repeated for a total of 3 times)
   1. Playlist-Making: participants must select 3 song clips from a list of 10
      1. Participants are told that they should create playlists for use within a social situation (i.e., “to listen to with friends during a virtual get-together later this week”)
   2. Playlist-Listening: participants must listen back to their (3) song clip selections shuffled with (3) additional randomly selected song clips
      1. ALG: Participants informed that random selections were added by ALG
      2. FP: Participants informed that random selections were added by [randomly generated name of] FP
5. Recognition Task
   1. In each trial a song clip from Playlist-Making paradigm is played back one-by-one; participant must identify whether they, FP/ALG, or neither selected it for any of the (3) prior playlists
      1. Practice Round: trials 1-5, inclusive
      2. Test Round (stored data): trials 5-30, inclusive
6. Self-Report Items (Post):
   1. Inclusion of Other in Self (i.e. IOS; Aron et al., 1992)
   2. Interpersonal Reactivity Index (i.e. IRI; (Davis, 1980, 1983)
   3. Single-Item measure for perceived stress* (Littman et al., 2006)

*Not included in analysis

## Questionnaire and Self-Report Items

*(Catalogued with respect to the above outline)*

1a. Which of the following best describes you?

1: nonmusician

2: music-loving nonmusician

3: amateur musician

4: serious amateur musician

5: semiprofessional musician

6: professional musician

1b. How many hours a day do you spend listening to music?

1: <1 hour

2: 1-3 hours

3: 3-5 hours

4: 5-7 hours

5: >7 hours

1c. How familiar are you with hip-hop music?

1: not at all familiar

2: slightly familiar

3: somewhat familiar

4: moderately familiar

5: extremely familiar

1d. (1) Does this statement apply to you? "Having a similar taste in music often helps me relate better to my peers"

1: not at all

2: a little bit

3: to some extent

4: quite a bit

5: very much

1d. (2) Does this statement apply to you? "I am able to make more friends when we like the same type of music"

- 1: not at all
- 2: a little bit
- 3: to some extent
- 4: quite a bit
- 5: very much


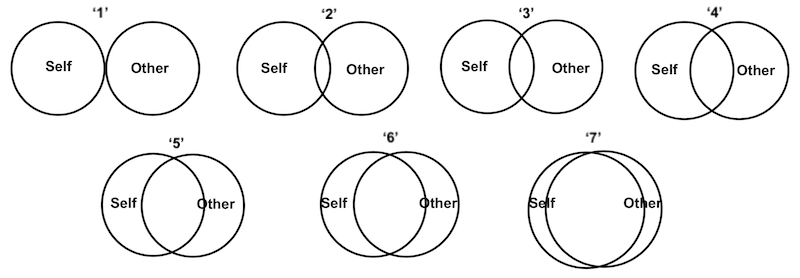


6a. Please use your keyboard (keys 1-7) to select one of the above pictures.

*For participants assigned to ALG condition:*

Which picture best describes your relationship with MusicBot, the recommendation algorithm?

*For participants assigned to FP condition:*

Which picture best describes your relationship with [fake_partner_name] the participant you collaborated with when making the previous playlists?

6b. To what extent does the following statement describe you:

I sometimes try to understand my friends better by imagining how things look from their perspective. (from 1 for “does not describe me” to 5 for “describes me very well)

# Supplementary Multimedia

## Supplementary Audio: Song Clip Stimuli

Zip folder containing all sound clip stimuli used in the online experiment. Each audio file is a ten-second clip of an instrumental hip-hop beat with the first 500 ms and last 500 ms linearly faded in and out, respectively, in order to control for immediate recognition of song clips during recognition task. [Audio quality has been downgraded (via wav to mp3 conversion) to allow for upload on Frontiers portal (30 MB size limit).]

## Supplementary Videos: Recognition Task Example Videos

Two videos (<=1 min. duration) illustrating the experimenter-designed Memory Recognition Task, showing the first several (approx. 10) trials for a mock participant. Each video depicts that with which a participant either in the algorithm (ALG) condition or in the fake partner (FP) condition would be prompted.
